# Endophenotype Network Models: Common Core of Complex Diseases

**DOI:** 10.1038/srep27414

**Published:** 2016-06-09

**Authors:** Susan Dina Ghiassian, Jörg Menche, Daniel I. Chasman, Franco Giulianini, Ruisheng Wang, Piero Ricchiuto, Masanori Aikawa, Hiroshi Iwata, Christian Müller, Tania Zeller, Amitabh Sharma, Philipp Wild, Karl Lackner, Sasha Singh, Paul M. Ridker, Stefan Blankenberg, Albert-László Barabási, Joseph Loscalzo

**Affiliations:** 1 Center for Complex Networks Research and Department of Physics, Northeastern University, Boston, MA, USA; 2 Center for Cancer Systems Biology, Dana-Farber Cancer Institute, Boston, MA, USA; 3 Department of Theoretical Physics, Budapest University of Technology and Economics, Budapest, Hungary; 4 Division of Preventive Medicine, Brigham and Women’s Hospital and Harvard Medical School, Boston, MA, USA; 5 Informaton Systems, Brigham and Women’s Hospital, Boston, MA, USA; 6 Department of Medicine, Brigham and Women’s Hospital, Harvard Medical School, Boston, MA, USA; 7 Center for Interdisciplinary Cardiovascular Sciences, Brigham and Women’s Hospital, Harvard Medical School, Boston, MA, USA; 8 University Heart Center Hamburg, Clinic for General and Interventional Cardiology, Hamburg, Germany; 9 German Center for Cardiovascular Research (DZHK), Partner site Hamburg/Lübeck/Kiel, Hamburg, Germany; 10 Channing Division of Network Medicine, Department of Medicine, Brigham and Women’s Hospital, Harvard Medical School, Boston, MA, USA; 11 Preventive Cardiology and Preventive Medicine, Dept. of Medicine 2, University Medical Center Mainz, Mainz, Germany; 12 Clinical Epidemiology, Center for Thrombosis and Hemostasis, University Medical Center Mainz, Mainz, Germany; 13 Institute for Clinical Chemistry and Laboratory Medicine, University Medical Center Mainz, Mainz, Germany; 14 Center for Network Science, Central European University, Budapest, Hungary

## Abstract

Historically, human diseases have been differentiated and categorized based on the organ system in which they primarily manifest. Recently, an alternative view is emerging that emphasizes that different diseases often have common underlying mechanisms and shared intermediate pathophenotypes, or *endo(pheno)types*. Within this framework, a specific disease’s expression is a consequence of the interplay between the relevant endophenotypes and their local, organ-based environment. Important examples of such endophenotypes are inflammation, fibrosis, and thrombosis and their essential roles in many developing diseases. In this study, we construct endophenotype network models and explore their relation to different diseases in general and to cardiovascular diseases in particular. We identify the local neighborhoods (module) within the interconnected map of molecular components, i.e., the subnetworks of the human interactome that represent the *inflammasome, thrombosome,* and *fibrosome*. We find that these neighborhoods are highly overlapping and significantly enriched with disease-associated genes. In particular they are also enriched with differentially expressed genes linked to cardiovascular disease (risk). Finally, using proteomic data, we explore how macrophage activation contributes to our understanding of inflammatory processes and responses. The results of our analysis show that inflammatory responses initiate from within the cross-talk of the three identified endophenotypic modules.

A limited number of key endophenotypes are common to all diseases. Most notable among them are inflammation, thrombosis, and fibrosis^1^. These endophenotypes reflect mechanisms that facilitate the organism’s adaptation to injury. Each has acute and resolving phases. The common goal of these underlying responses is to restore normal organ and organism function. In as much as these endophenotypes evolved to promote healing from acute injury, their implications for chronic injury or disease likely had a lesser role, if any, on their genetic selection. As a result, chronic overexuberant inflammatory, thrombotic, or fibrotic responses can yield organ impairment and adverse long-term effects that outweigh the acute benefits they provide^2^,^3^,^4^,^5^.

Inflammation, thrombosis, and fibrosis are pathologically linked^6^: inflammation can induce (accelerate) thrombosis, thrombosis can induce inflammation^7^,^8^, and fibrosis can result from resolving inflammation and thrombosis^9^. For these reasons, we explored the joint molecular network determinants of these endophenotypes, in particular aiming to identify those molecular subnetworks or mediators that are common to all, as well as those that are distinctive for each. In this way, we can define the determinants of the interplay among these common endophenotypes, as well as the determinants of heightened or deficient responses in them.

A complex cascade of molecular interactions occurs during inflammatory, thrombotic, and fibrotic processes, many of which remain poorly understood. Several molecules and cell types play a crucial role in these processes and exert their function through a network of interactions. Therefore, in this study we explore (a) network models of inflammation, thrombosis, and fibrosis; (b) their biological and topological crosstalk; and (c) the role of macrophages as central cellular mediators of these endophenotypes.

## Results

### Constructing the inflammasome, thrombosome, and fibrosome

**Figure 1:**
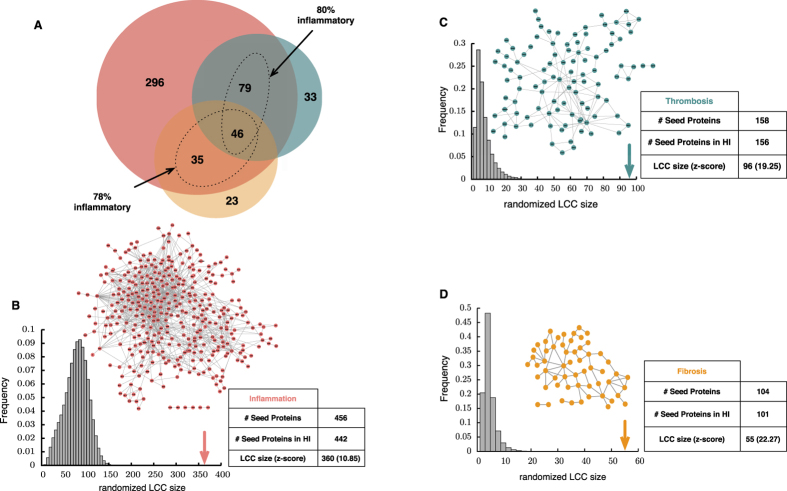
Topological characteristics of seed genes within the Human Interactome. (**A**) Venn diagram of inflammatory (red), thrombotic (blue), and fibrotic (orange) seed genes. (**B**–**D**) correspond to subgraphs of the human interactome containing inflammatory, thrombotic, and fibrotic seed genes, respectively. These genes form a giant connected component, suggesting the existence of a local network neighborhood enriched with inflammatory, thrombotic, and fibrotic genes. The randomized distribution of the LCC size is shown in the histograms. For the effect of literature bias, see SI.

We start our analysis by assembling a set of genes with established association (*seed genes*) with inflammation, thrombosis, and fibrosis from the literature (see Methods). In order to obtain genes with high-confidence association, we used two filtering criteria: (a) genes whose association has been reported in at least two publications; and (b) genes that are expressed in tissues related to cardiovascular diseases. The final numbers of seed genes for inflammation, thrombosis, and fibrosis were 456, 158, and 104, respectively (Table 1). As expected, the three gene sets show significant overlap; for example, 80% (*p*-value = 1.50 × 10^−161^; Fisher’s exact test) and 78% (*p*-value = 3.58 × 10^−104^) of the genes associated with thrombosis and fibrosis, respectively, are also associated with inflammation (Fig. 1A). The list of seed genes is available in Data file S1.

For an independent biological evaluation of the compiled seed gene lists, we tested for association between candidate functional single nucleotide polymorphisms (SNPs) mapping to each seed gene and selected established cardiovascular biomarkers, C-reactive protein (*CRP)*, fibrinogen, soluble intercellular adhesion molecule (*ICAM),* as well as a clinical vascular pathophenotype, venous thromboembolism (*VTE*) (see Methods and Supplementary Fig. 1). Although curated seed genes are not necessarily expected to overlap with genetic associations meeting genome-wide significance (P-value < 5 × 10^−8^), we observed that for all four validation sets (CRP, fibrinogen, ICAM and VTE), inflammation and thrombosis seed genes carry a larger fraction of low *p*-value as compared to other genes in the network. We observed a similar effect for fibrosis seed genes with respect to CRP, ICAM, and VTE, but not fibrinogen (Supplementary Fig. 1).

To identify the sub-networks corresponding to the three endophenotypes, we compiled the human interactome (HI) from several data sets containing physical binary interactions among molecular components. These datasets include regulatory, binary, kinase-substrate, metabolic, liver-specific, and protein complex-based interactions (see Supplementary Material). The final HI consisted of *N* = 13,681 proteins (nodes) and *M* = 144,414 interactions between them (edges).

Considerable evidence suggests that genes associated with complex diseases are not randomly scattered within the HI but tend to interact with each other in specific network neighborhoods, or *disease modules*^10^,^11^,^12^. The same phenomenon is found for the seed genes of the three endophenotypes: their seed genes form connected subgraphs whose sizes are significantly larger than expected by chance for randomly distributed genes (Fig. 1B–D and Table 1). To estimate the extent to which literature biases in our HI may be responsible for the observed clustering, we repeated the analysis using a high-throughput (yeast two-hybrid) interactome, and confirmed that the observed clustering, indeed, reflects the existence of modules responsible for these endophenotypes (see Supplementary Material effects of biased studies in the Human Interactome on disease gene clustering).

**Figure 2:**
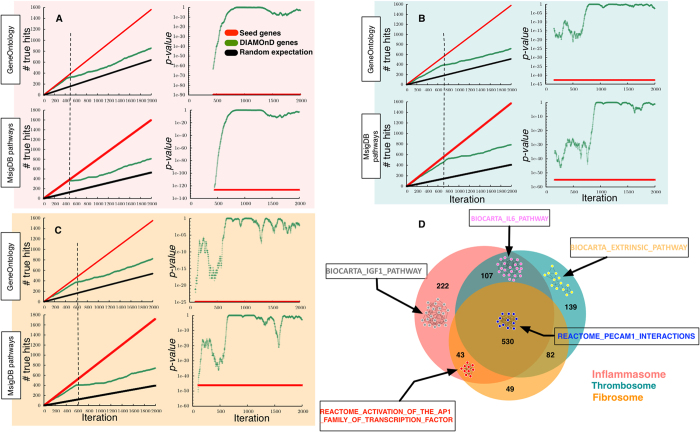
Biological validation of the detected DIAMOnD genes. Panels correspond to validating DIAMOnD genes of inflammation (**A**), thrombosis (**B**), and fibrosis (**C**), respectively (red lines, seed genes; green lines, DIAMOnD genes; black lines, randomly selected genes). Validation is assessed with respect to GeneOntology and MSIgDB pathways. As the DIAMOnD genes are iteratively added to the neighborhood, the *p*-value of enrichment increases with a clear jump to non-significant values (*p-*value ~ 1) at the indicated iteration. Therefore, we use the suggested iteration steps to define cutoffs for the methodology, and thereby identify the size limit of the underlying associated module. We chose 450, 700, and 600 first identified DIAMOnD nodes to form the inflammasome, thrombosome, and fibrosome modules, respectively. (**D**) Venn diagram of the inflammasome, thrombosome, and fibrosome genes. The fully embedded pathways within detected modules have been found in inflammasome-specific proteins, thrombosome-specific proteins, overlapping proteins in the inflammasome and thrombosome, and overlapping proteins in all three modules.

**Figure 3:**
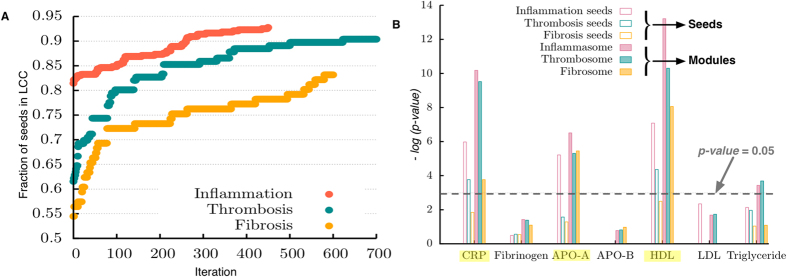
Topological properties and robustness of the endophenotypic modules. (**A**) Previously disconnected seed genes are now connected to each other through detected DIAMOnD genes. The inflammasome, thrombosome, and fibrosome modules so-constructed allow 93%, 90%, and 83% of seed genes to become part of the LCC, respectively. (**B**) Enrichment of seed genes and modules with differentially expressed genes in subjects with a significant cardiovascular risk factor burden.

We used the seed gene clusters in the interactome as a starting point to explore the molecular mechanisms of the respective endophenotypes in the broader context of *disease-associated endophenotype modules,* i.e., sub-networks associated with inflammation, thrombosis, and fibrosis. To identify these neighborhoods of endophenotype proteins, we used the DIseAse MOdule Detection (DIAMOnD) method that iteratively expands the seed gene neighborhood by adding proteins with a significant number of connections to the seed gene pool^12^. In principle, the method ranks all proteins in the network. To identify the boundary of each endophenotype module, we, therefore, considered their biological relevance with additional biological evidence (see Methods). We found that approximately the first 450, 700, and 650 DIAMOnD genes show a clear and significant biological association with inflammatory, thrombotic, and fibrotic seed genes, respectively (Fig. 2A–C). DIAMOnD genes, together with the seed genes, form three endophenotype modules that we call the *inflammasome, thrombosome,* and *fibrosome*, containing 902, 858, and 704 proteins, respectively. Moreover, through the addition of 450 DIAMOnD proteins, 93% of inflammatory seed proteins are integrated into a connected component (LCC) (Fig. 3A). Therefore, the additional DIAMOnD proteins allow for the integration of previously disconnected seed proteins into the (largest) connected component of the modules. The resulting modules are robust towards small variations in the initial seed gene set. DIAMOnD methodology predicts a robust outcome with almost complete overlap when removing a random gene from the original set of seeds (Supplementary Fig. 2, see SI for details on the *N*-1 analysis).

The three modules have a large common core of 530 proteins (Fig. 2D). The thrombosome and inflammasome show significant (*p-*value < 10^−324^; see Methods) overlap of 637 genes (Jaccard index *J* = 0.57). This region could be further investigated for inflammation-induced thrombotic pathways^13^. It is known, for example, that inflammation inhibits natural anticoagulant pathways and fibrinolytic activity as well as increases procoagulant factors, thereby increasing the (net) thrombotic response.

Interestingly, the overlap between the modules is more significant than the overlap between the seed genes, suggesting that these endophenotypes are truly in the same neighborhood of the interactome. Further pathway analysis of the genes within the modules identified five fully embedded pathways: IL6, IGF1, extrinsic prothrombin activation, AP1 family of transcription factors, and PECAM1. The pathways presented are exclusive to the region shown. In other words, the figure represents two pathways enriched in the overlap of three modules, as well as an inflammation-specific pathway, a thrombosis-specific pathway, and one pathway enriched in a region common to the inflammasome and thrombosome (Fig. 2D, Supplementary Table 1).

### The role of endophenotype modules in cardiovascular disease risk and other complex diseases

We next turned to an analysis of the identified modules with respect to (*a*) cardiovascular disease risk (an example of preclinical disease), and (*b*) their more general association with complex diseases.

To assess the potential role of the three endophenotypes for the risk of developing cardiovascular diseases, we analyzed gene expression data in monocytes from a cohort of 1,258 individuals^14^,^15^ (see Methods), comparing individuals at high risk of cardiovascular diseases (cases) to patients at low risk (controls). Quantitative biochemical risk factors measured in the population included CRP, fibrinogen, high-density lipoprotein cholesterol (HDL-C), low-density lipoprotein cholesterol (LDL-C), apolipoprotein-A (APO-A), apolipoprotein-B (APO-B), and triglycerides (see Methods). We found that the respective sets of differentially expressed genes exhibit a significant overlap with each other (Supplementary Fig. 3). All three endophenotypes are strongly enriched with CRP, HDL, and APO-A-associated genes, which affirms the results of a previous proteomics study reflecting the link between HDL and inflammation^16^. The inflammasome and thrombosome were additionally enriched with triglyceride-related genes (Fig. 3B, Supplementary Table 2). The link of lipid-associated genes with thrombosis confirms prior work^17^.

For a more general assessment of the role of the three endophenotypes in complex diseases other than cardiovascular diseases, we next analyzed their enrichment with disease proteins from a corpus of 299 diseases^18^. We found that, in total, the disease-genes associated with 156 (52% of) diseases significantly overlap with at least one of the three detected modules (Supplementary Table 3). Among these diseases, 67 are enriched in all three modules, while 11, 10 and 26 are inflammasome-, thrombosome-, and fibrosome–specific, respectively (Supplementary Table 4). These data support the notion that inflammation, thrombosis, and fibrosis are pathobiological endophenotypes common to many diseases.

In summary, we observed that the three detected endophenotype modules are highly enriched with known disease genes, in general, and, more specifically, with differentially expressed genes associated with cardiovascular risk factors. Hence, the detected subregions of the network, including the proteins and their intermolecular interactions, are likely to be of high biological importance and worth analyzing in the context of disease development.

### Topological properties of the endophenotype modules (central location of inflammatory and fibrotic genes)

Prompted by the strong enrichment of the endophenotype modules with genes associated with complex diseases and preclinical cardiovascular disease (cardiovascular risk factors), we explored whether this central role is also reflected in specific topological properties of the modules within the interactome.

**Figure 4:**
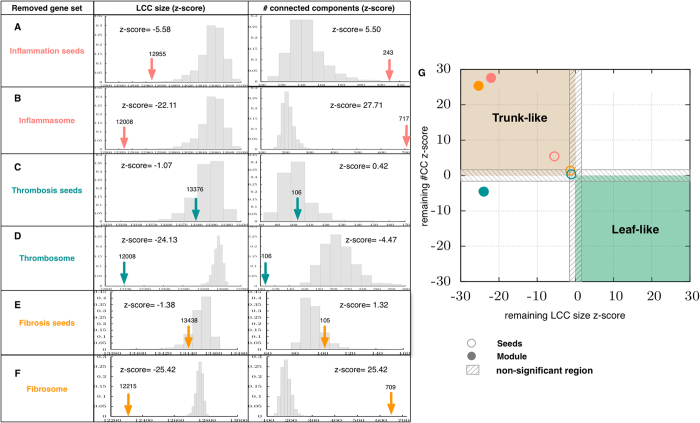
Tree analysis of seed genes and modules. Panels (**A**) through (**F**) show the observed size of the LCC and the number of connected components after removing the denoted gene sets. The observed parameter is compared to that of random expectation and a *z*-score is calculated. Panel G shows the phase diagram of *z*-score(CC) and z-score(LCC) of inflammation-, thrombosis-, and fibrosis-associated genes. As shown, the inflammasome, thrombosome, and fibrosome, as well as inflammatory seed genes, are highly essential for defining the clustered structure of the network.

To do so, we analyzed the extent to which the robustness and structural integrity of the network depend on these proteins using a “tree” analysis (see Methods), i.e., testing whether a set of nodes constitutes an essential backbone of the HI (“trunk” of the tree) or whether it is of secondary importance for the overall structure (“leaves”) (Supplementary Fig. 4). The results of this analysis on both seeds and module proteins show that inflammatory seeds and modules as well as the fibrosome are trunk-like and, thus, essential for the overall integrity of the network (with high *z-score(CC)* and *z-score(LCC))* (Fig. 4A–D,G). Note that these results cannot be attributed to high average degree and centrality alone. Furthermore, despite having higher average degree and betweenness centrality, thrombosis and fibrotic seed proteins are not trunk-like (Fig. 4). See supplementary material for a list of basic topological properties of these modules (Supplementary Table 5).

It is worth noting that thrombosis and fibrosis seeds are near-subsets of the inflammation seeds, i.e., ~80% of the seeds are inflammatory. However, only inflammation seeds are trunk-like. Similarly, although the inflammasome and thrombosome overlap significantly and are comparable in size, only the inflammasome shows trunk-like behavior. These notable distinctions (a) exclude the possibility that size might be responsible for this effect, and (b) indicate that the non-overlapping proteins are responsible for the observed differences in essentiality. Overall, we conclude that the enrichment of the inflammasome with different disease determinants is rooted in its topologically centered location within the HI.

### Functionality of detected endophenotype modules using macrophages

During inflammatory responses, monocytes differentiate into macrophages^19^, which appear to be a heterogeneous population. Differences among macrophage subpopulations reflect their gene expression pattern, protein levels, and functions. M(IFNγ) or M1 macrophages may play a key role in the acute phase of inflammation through the production of injurious molecules, whereas M(IL-4) or M2 cells may participate in tissue repair in a later phase.

Accumulating evidence from the literature suggests a role for pro-inflammatory macrophages in various aspects and stages of the development of cardiovascular diseases^20^,^21^. Several lines of evidence in humans have clearly associated the dominance of M1-like macrophages or activated circulating monocytes with cardiovascular risk factors [e.g., hyperlipidemia, diabetes^22^,^23^], plaque phenotype [e.g., unstable plaque^24^,^25^], or clinical events. The activated macrophage phenotype has typically been gauged by production of pro-inflammatory cytokines and chemokines, which IFNα typically induce in THP-1 cells^26^.

**Figure 5:**
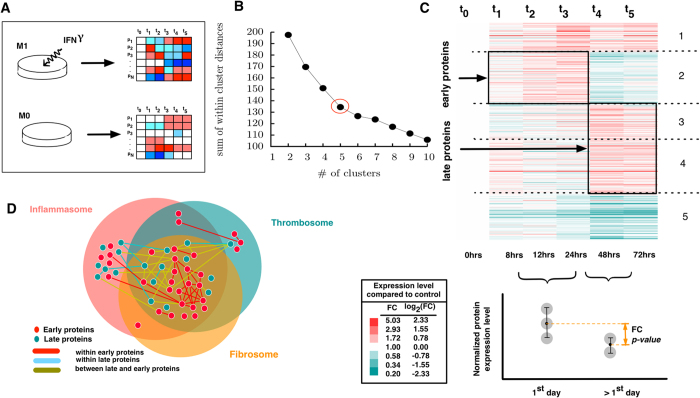
Detecting early and late proteins of inflammatory responses. (**A**) Schematic representation of inducing inflammatory stimulator to THP1 cells. (**B**) Sum of within cluster distances vs. number of clusters where *k* = 5 was found to detect optimal clustering. (**C**) Clusters formed by *k*-means clustering analysis of M1 macrophages where two boxes indicate late and early expressed protein. (**D**) Network representation of early and late proteins within detected endophenotype modules and the enrichment of early proteins within cross-talk region of the three endophenotypic modules.

In order to identify proteins that play a role in pro-inflammatory^27^,^28^ responses, we used two unbiased quantitative proteomic datasets generated from human THP-1 macrophage-like cells without (M0 (untreated)) or stimulated with INFγ (M(IFNγ) or M1^29^. Proteins were sampled at six time points up to 72 hours of stimulation (see Methods for data description and analysis). This experimental procedure yields a time series of protein abundance that can inform or suggest downstream causation (Fig. 5A).

There were 3,821 proteins with at least one interacting partner in the HI detected in both M1 and the baseline control, M0. Among these proteins, 447 overlap with endophenotype modules (*p-*valu*e* = *1.40* × 10^−15^). We refer to these 447 proteins as “ome-M1” proteins, indicating the detected proteins in both M0 and M1 that overlap with the three endophenotype modules, the inflammasome, the thrombosome, and the fibrosome. We observed that the functional annotations of the ome-M1 proteins differ significantly from the rest of the detected proteins (Supplementary Fig. 5, see Methods).

As we are interested in finding proteins responsive to inflammatory stimuli, we studied the proteins’ abundance in M1 relative to M0 (where proteins are not induced and their abundance varies normally). Therefore, we first calculated the fold change of protein abundances at each time point. We did this by dividing the protein abundances in M1 by M0. Next, we identified subgroups of enhanced and suppressed proteins by applying a *k*-means clustering on the time series of fold changes for ome-M1 proteins (See Methods). The changes in sum of within-cluster distances (*sw*) of protein levels with respect to the number of clusters suggests *k* = *5* clusters as an optimal number of clusters (Fig. 5B, elbow method). The first (last) identified cluster represents a set of proteins with high (low) relative abundance throughout the measurement time (Fig. 5C). Cluster 2 represents a subset of proteins in which protein abundance is higher than the M0 baseline during the first day and decreases thereafter. At the same time, clusters 3 and 4 together represent two subsets of proteins that are highly expressed only after the first day of activation with IFN*γ*. We refer to these two subgroups as early and late proteins where early proteins have an elevated relative abundance within the first days and decreased levels thereafter, and late proteins are unaffected within the first day and increase their expression after 24 hrs.

This observation suggests that the high abundance of early proteins on the first day is mechanistically linked to the abundance of late proteins. This observation is also consistent with the connectivity patterns among early and late proteins within the interactome: Each late or early protein has *k*_in_ interactions with the other proteins within its own group and *k*_out_ interactions with the proteins of the other group. We find that early proteins tend to interact with late proteins more than they do with themselves. In contrast, late proteins tend to interact with each other more than they interact with early proteins. An early protein has an average *k*_in_ of 3.33 and an average *k*_out_ of 8.24, whereas a late protein has an average *k*_in_ of 9.71 and an average *k*_out_ of 4.71. This observation suggests that early proteins are responsible for triggering late proteins, while downstream, triggered late proteins tend to interact with each other.

To define a high confidence set of early and late proteins, we compared the average abundance levels of proteins within and after the first 24 hrs (Fig. 5D) and selected those that satisfy three different confidence criteria (See Methods for more details). Early and late proteins, while separated, are interconnected within the modules and, thus, directly influence each other. A list of the top 20 pathways enriched by early and late proteins characterized by the most stringent confidence criterion (See Methods) can be found in Table 2. Supplementary Tables 6 and 7 list the same properties for proteins characterized by less stringent criteria. These early and late proteins are interconnected within the modules (Fig. 5E) and, thus, likely affect each other. That they do so is supported by a thorough review of the 33 early proteins and 18 late proteins in Table 2 for those for which there is literature evidence of a mechanistic association as a validation of the network approach. Among the early proteins, a minority–i.e., five–show mechanistic (binding or pathway-dependent) links to the triggering of late protein expression by published experimental evidence. These include CARD9, which can trigger IL1B^30^ and CASP7^31^; PARP1, which can induce NAMPT^32^ and TRADD^33^; CD36, which can trigger IRAK1^34^; PRKDC, which can induce NAMPT^35^; and HSPB1, which can also induce TRADD^36^. Interestingly, the induction of CASP7 can lead to a reduction in PARP1^37^. The interrelationships between early and late proteins of pathways that affect interleukin signaling, TNF signaling, NAD synthesis, and caspase activation are clear from this analysis and highlight those molecular features conventionally viewed as ‘inflammatory’ as central to late-appearing protein responses that also play a key role in thrombosis and fibrosis.

Unlike late proteins, early proteins are significantly located within the area of overlap among the thrombosome, inflammasome, and fibrosome modules (*p*-value = 0.01) (Fig. 5E). This finding suggests that an early core response common to all endophenotypes disseminates throughout the endophenotype network to the more distinct endophenotypes at later times. A list of all genes in the cross-talk region is provided in a Data file S2.

## Discussion

Beginning with high confidence literature-curated seed genes and using the DIAMOnD methodology, we detected three sub-regions within the HI associated with inflammatory, thrombotic, and fibrotic responses. These highly overlapping regions are significantly enriched with several disease determinants, including: (a) disease genes associated with more than 50% of the compiled complex diseases, and (b) differentially expressed genes associated with cardiovascular risk factors (i.e., preclinical disease). Separately, we found IL6, IGF1, extrinsic prothrombin activation, the AP1 family of transcription factors, and PECAM1 pathways to be fully embedded within these modules. The three endophenotypes are not only of interest in terms of functional enrichment, but also lie within a topologically important region of the HI. We showed that proteins belonging to the inflammasome and fibrosome are highly essential for maintaining the overall structure and integrity of the network.

To study further the rather large number of proteins in the predicted modules, we dissected them into functional subgroups. As proteins function through a cascade of interactions among cellular components, it is important to be able to map this biological and topological information to a potential molecular mechanism and find the most relevant underlying pathways. We, therefore, divided the genes within the modules into different subgroups, each of which having a certain role in inflammatory processes. These subgroups are defined based on the protein clusters with similar expression pattern towards the inflammatory cytokine (INFγ). Detailed analysis of module response to INFγ led us to observe four significantly distinctive protein abundance patterns belonging to: (a) expressed proteins, (b) silent proteins, (c) early proteins, and (d) late proteins. Present (silent) proteins show an elevated (decreased) level of abundance throughout the course of 72 hrs after INFγ exposure. Early proteins manifest an elevated abundance during the first day, while late proteins show low abundance during the first day and are increased in abundance thereafter. Our observations suggest that the common underlying mechanism of many inflammatory-driven complex diseases resides in the common core of the endophenotype modules detected in this work.

We studied the obvious limitations of the interpretations drawn from our observations, such as the biased and incomplete nature of the HI maps. Based on our analysis, the initial observations leading to the detection of the endophenotypic modules is robust and holds in unbiased maps, and, thus, are expected to improve with increasing coverage of the existing maps. The endophenotypic modules as well as their region of cross-talk detected in this work merit more clinical attention in the context of the pathobiology and treatment of inflammatory-driven phenotypes.

In addition, the proteins within the detected regions and their interactions warrant further study in the search for proposing molecular mechanisms and candidate drug targets for specific diseases.

## Methods

### Curation of disease genes associated with inflammation, thrombosis, and fibrosis

We used HuGe Navigator (http://www.hugenavigator.net) to retrieve genes with established associations with inflammation, thrombosis, and fibrosis. HuGE Navigator is a continuously updated and publicly available knowledge base that retrieves genes associated with a phenotype of interest by first parsing PubMed articles and subsequently manually reviewing the results by experts.

### Tissue specificity

To obtain disease-gene associations of higher confidence, we restricted the seed genes to those present in at least one cardiovascular disease-specific tissue using gene expression data from 79 human tissues^38^. The specific tissues considered include: monocytes, vascular smooth muscle cells, endothelial cells, T-cells, and hepatocytes. We consider a gene to be expressed in a tissue if its expression level in the healthy state meets one of the following criteria:

1. Expression level of 200 mRNA counts or higher in the specified tissue.
2. The expression level in the specified tissue is significantly higher than the expression profile across all tissues. For this analysis we used a modified *z*-score > 1.6 defined by^39^:

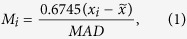


where, *MAD* is the median absolute deviation and 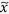 denotes the median.

### Human Interactome

We only consider direct physical interactions among molecular components with reported experimental evidence. For this purpose, we consolidated several data sources including regulatory interactions^40^; binary interactions containing high-throughput datasets^41^,^42^,^43^,^44^ with binary interactions from IntAct^45^ and MINT^46^ databases; literature-curated interactions from IntAct, MINT, BioGRID^47^, and HPRD^48^; metabolic enzyme-coupled interactions^49^; protein complexes^50^; kinase network^51^; signaling interactions^52^; and liver-specific interactions^53^. The resulting network has a power-law degree distribution^54^. For more information, see Supplementary Material.

### Genetic Association

Genotype data for candidate functional genetic polymorphisms were collected in the Women’s Genome Health Study (WGHS)^55^ using the Human Exome BeadChip platform v.1.1 (Illumina, San Diego) and reduced to genotype calls as described^56^. We have used this data set as it is derived from the largest genome-wide study available to us. The genetic markers on this platform predominantly cause non-synonymous substitutions, splice site disruptions, or other known functional changes allowing relatively unambiguous assignment to genes that are affected by their molecular consequences. In total, there were 22,516, 22,390 and 22,411 WGHS participants of verified European ancestry with genotype data and plasma measures of C-reactive protein (CRP), sICAM1, and fibrinogen respectively. Similarly, there were 526 cases of incident venous thromboembolism (VTE) compared with 21,479 unaffected WGHS participants with genotype data. SNPs were tested for association using linear (plasma biomarkers, log-transformed and residualized for age and population eigenvectors, if needed) or age- and eigenvector-adjusted logistic (VTE) regression. For each gene, a gene-wide p-value corrected for multiple testing was computed by the Šidák method applied to the minimum p-value among the SNPs mapping to each gene and having minor allele frequency of at least 0.0005.

### LCC significance

The significance of the clustering of a given set of nodes is obtained by comparing the observed LCC size with the size expected for randomly distributed nodes of a set of the same size obtained from 10^6^ simulations. The resulting *z-score* is defined:


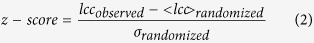


where *lcc* is the size of largest connected component, and <*lcc>*_*randomized*_and *σ* are the average size and standard deviation, respectively, of the largest connected components across all randomized sets.

### Significance of module overlap

Part of the high overlap of the modules stems from common seed genes in the three endophenotypes. Considering that the seed genes represent established knowledge, calculating the significance of the module overlap reduced to calculating the significance of the overlap between the added (DIAMOnD) genes. We calculate the significance of inflammasome and thrombosome overlap using the following methods:

(a) First, we consider that 450 (700) detected DIAMOnD nodes with respect to inflammation (thrombosis) could have been selected from any nodes in the network. We calculate the *p-*value using Fisher’s exact test, resulting in a *p-*value < 10^−324^.

(b) In practice, the detected nodes cannot be selected from anywhere in the network. Rather, they are iteratively added based on their connectivity patterns to seed nodes. To factor this principle into the analysis, we assume that detected nodes can be selected from first neighbors of seeds only. We further limit this pool of candidate nodes by taking those that are first neighbors of both inflammation and thrombosis seeds. This selection process will underestimate the resulting significance. Fisher’s exact test yields a *p-*value < 10^−324^.

(c) With the same approach, we found the overlapping significance of *p*-value < 10^−324^ for both inflammasome-thrombosome, and thrombosome-fibrosome pairs, respectively.

### Biological validation and module size estimation

We used Gene Ontology and MSIgDB^57^ pathways as follows:

(a) MSIgDB pathways: From the MSIgDB database, we retrieved the biological pathways significantly enriched with seed genes (FDR corrected). Next, we show that these pathways are also statistically highly enriched with DIAMonD genes. Figure 2 shows the number of DIAMOnD genes that belong to these sets of pathways as a function of DIAMOnD iteration and their corresponding *p*-values.

(b) Gene Ontology (GO): In the same fashion, we extracted GO terms [ http://www.geneontology.org/, downloaded April, 2016] significantly annotated for the seed genes and show that DIAMonD genes are significantly annotated for the same GO terms.

### Pathway enrichment

We performed pathway enrichment analysis for four different regions: (a) inflammasome, (b) thrombosome, (c) fibrosome, and (d) crosstalk. Supplementary Table 1 shows fully embedded pathways in specific regions of the modules.

### Tree analysis

To assess whether a given set of nodes is essential for the integrity of the network, we remove them from the network and measure two parameters: (a) the number of remaining connected components (islands), and (b) the size of the remaining LCC. Next, we compare the results to expected values of these measures as follows:

(a) Randomly select the same number of nodes from the network.

(b) Remove these nodes from the network.

(c) Measure parameters as introduced above (a and b).

(d) Repeat steps (a–c) 10^6^ times to produce a randomized distribution.

(e) Calculate a *z*-score for the actual observation.

Highly positive (negative) z-scores of the LCC size (number of connected components) reflect a central location of the respective nodes. A group of nodes whose removal results in a significantly higher number of connected components (*z-score(CC)* > 1.6) with a much smaller LCC (*z-score(LCC)* < −1.6) is considered essential for the integrity of the HI, i.e., is trunk-like. By contrast, nodes whose removal leads to a significantly decreased number of connected components (*z-score(CC)* < −1.6) and a larger LCC (*z-score(LCC)* > 1.6) are considered non-essential, i.e., are leaf-like (Supplementary Fig. 4).

As we compare the observed *lcc* size to over 10^5^ randomizations, the randomized pool distribution approaches a normal distribution and, thus, using a *z-score* is a sensible choice for calculating the significance of deviation from a random distribution. In a normal distribution, a *z-score* of 1.6 is equivalent to a *p-value* of 0.05, a widely used significance threshold. Importantly, although we use a threshold of 1.6 for *z-score* significance, our results show that the *z-scores* associated with clustering of the inflammation, thrombosis, and fibrosis seed genes are 10.85, 19.25, and 22.27, respectively. Thus, setting an even more stringent threshold would also confirm the significance of the observed *lcc*.

### Cardiovascular risk and differentially expressed genes

We used gene expression data derived from the population-based Gutenberg Health Study (GHS). The dataset consists of mRNA counts of the Illumina HT-12 v3 BeadChips (*n* = 1,285). Analyses were conducted at the University Heart Center, Hamburg, Germany.

We analyzed the data to retrieve differentially expressed genes associated with cardiovascular risk factors. The sample sizes were selected so that the biomarker levels are consistent with the recommended effect size (low and high risk ranges) from the literature^58^,^59^,^60^. Therefore, we defined cases and controls as individuals with the top and bottom 25% of the risk factor level distribution. The case and control sample sizes are listed in Supplementary Table 8. To derive the differentially expressed genes, we performed a non-parametric Mann-Whitney U-test. Supplementary Fig. 3 shows the Venn diagram of differentially expressed genes with respect to different risk factors.

We showed that the inflammation, thrombosis, and fibrosis gene (networks) are significantly enriched with differentially expressed genes of CRP, HDL-C, APO-A, and triglyceride modules. There were only 9 and 3 differentially expressed genes associated with APO-B and LDL-C. Therefore, due to lack of statistical power, we observed non-significant enrichment of module proteins with those genes. Surprisingly, neither the seeds nor detected modules were enriched with fibrinogen genes.

### THP-1 cell culture experiments and proteomics

We first treated the human monocytoid cell line THP-1 with PMA for 48 hours to promote their differentiation into macrophage-like cells. As the specific molecular mechanisms by which INFγ promotes M1 responses (e.g., pro-inflammatory cytokine production) have been extensively studied, we chose INFγ as the M1-polarizing activated macrophage stimulus. Supplementary Fig. 6 demonstrates that INFγ treatment in THP-1 cells induced the potent pro-inflammatory molecules, TNFα and IL-1β, commonly used as markers of M1 activated macrophages. THP-1 cells (ATCC) were then incubated without (M0) or with 10 ng/ml INFγ for 72 hours (M1). Cells were collected from each time course condition at six time points –0, 8, 12, 24, 48 and 72 hours –for subsequent protein isolation, proteolysis, fractionation using isoeletric focusing (OFF-gel, Agilent)^61^,^62^ and tandem mass tagging [TMT-6plex, Pierce] as described previously^63^. The peptides were analyzed by an LTQ-Orbitrap Elite model (Thermo Scientific) coupled to an Easy-nLC1000 HPLC pump (Thermo Scientific). The top 20 precursor ions (within a scan range of 380–2000 *m/z*, resolution set to 120 K) were subjected to higher energy collision-induced dissociation (HCD, collision energy 40%, isolation width 3 m/z, dynamic exclusion enabled, and resolution set to 30 K) for peptide sequencing (MS/MS). The MS/MS data were queried against the human Uniprot database (downloaded on March 27, 2012) using the SEQUEST search algorithm via the Proteome Discoverer (PD) Software package (version 1.3, Thermo Scientific) using a 10 ppm tolerance window in the MS1 search space, and a 0.02 Da fragment tolerance window for HCD. Methionine oxidation and 6-plex TMT tags (Thermo Scientific) were set as variable modifications, and carbamidomethylation of cysteine residues was set as fixed modification. The peptide false discovery rate (FDR) was calculated using Percolator provided by PD: the FDR was determined based on the number of MS/MS spectral hits when searched against the reverse, decoy human database^64^,^65^. Peptides were filtered based on a 1% FDR. Peptides assigned to a given protein group, and not present in any other protein group, were considered as unique and quantified for the protein (PD Grouping feature).

Proteomic analysis revealed that 4,680 (4,589) proteins were detected (with at least 2 unique peptide ID and a unique gene ID) in the M0 (M1) condition, among which 4,278 (4,188) proteins were found to have at least one interacting partner in the consolidated HI.

In order to explore protein abundance changes in response to inflammatory stimuli, we restricted our study to the proteins whose levels were measured in both M0 and M1 conditions. We found that of the 4,143 proteins found in both datasets, 447 reside within the detected endophenotype modules (*p*-value = 1.13 × 10^−9^).

Next, we observed that the M0 and M1 proteins residing in the endophenotype modules (ome-M proteins) are functionally and significantly different from those outside of the module. To do so we proceeded as follows:

(a) We store the pathways enriched by ome-M proteins in a pathway array 
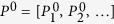
.

(b) From all proteins detected in M0 and M1 condition, we randomly select the same number of ome-M proteins.

(c) We store the pathways enriched by these randomly selected proteins in 
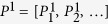
.

(d) We repeat steps (b) and (c) 1000 times to find 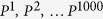
enriched pathway arrays.

(e) We calculate the Jaccard similarity of all pairwise combinations of the 1000 pathway arrays.

(f) Next, we calculate the Jaccard similarity of *P*^0^ with every of the 1000 pathway arrays.

The distribution of Jaccard similarities calculated in step (e) and (f) are shown in gray and red respectively in Supplementary Fig. 5. Our observation shows that detected proteins that reside in the modules functionally and significantly differ from other detected proteins outside the modules. Therefore, we limit our further studies to these ome-M proteins.

To identify the early and late proteins within the ome-M proteins, we have studied the average FC of protein abundance before and after 24 hrs. As the FC threshold is an arbitrary choice, we have included three levels of confidence for identifying early and late proteins. The criteria for these three levels of confidence include: (a) *p-*value < 0.05, (b) FC > 1.5, and (c) *p-*valu*e* < 0.05 and FC > 1.5. Table 2 shows the early and late proteins identified by the most stringent criterion (c).

### Clustering Analysis and visualization

For performing k-means clustering, we used Cluster 3.0, and for viewing the heatmap, we used Java TreeView.

## Additional Information

**How to cite this article**: Ghiassian, S. D. *et al.* Endophenotype Network Models: Common Core of Complex Diseases. *Sci. Rep.* **6**, 27414; doi: 10.1038/srep27414 (2016).

## Supplementary Material

Supplementary Information

Supplementary Dataset 1

Supplementary Dataset 2

## Figures and Tables

**Table 1:** Genes associated with endo-phenotypes, on the human interactome.

**Endo-phenotype**	**#HuGENet genes**	**# Seed Genes**	**# Seed Genes in HI**	**LCC size**	**z-score**
Inflammation	1679	456	442	360	10.85
Thrombosis	603	158	156	96	19.25
Fibrosis	621	104	101	55	22.27

**Table 2:** Topological and biological properties of early and late proteins characterized by confidence level criterion (c): FC > 1.5 and *p*-value < 0.05.

**FC > 1.5 p<0.05**	**Statistics**	**Proteins**	**Top 20 enriched pathways**
Early proteins	#proteins = 33M = 26LCC size = 14<k> = 81.03<kin> = 1.57<kout> = 0.64pin, out = 0.01	STMN1, VAV3, ITGA3, CARD9, GNA12, IFNGR1, RASA1, PARP1, CD36, SCARB1, CSNK2A1, PTGS1, CD22, TNPO1, DHFR, PEBP1, GPX1, AKT2, PRKDC, CD9, LRPPRC, HSPB1, TOP2A, CLTC, CABIN1, CD58, CCNB1, CALM1, CDK1, CDK9, CDK7, CSK, RPS27A	REACTOME_HEMOSTASISREACTOME_FORMATION_OF_PLATELET_PLUGBIOCARTA_PTC1_PATHWAYBIOCARTA_SRCRPTP_PATHWAYREACTOME_PLATELET_ACTIVATIONREACTOME_CYCLIN_A1_ASSOCIATED_EVENTS_DURING_G2_M_TRANSITIONBIOCARTA_CELLCYCLE_PATHWAYBIOCARTA_G2_PATHWAYKEGG_HEMATOPOIETIC_CELL_LINEAGEREACTOME_E2F_MEDIATED_REGULATION_OF_DNA_REPLICATIONREACTOME_G1_S_TRANSITIONKEGG_CELL_CYCLEREACTOME_E2F_ENABLED_INHIBITION_OF_PRE_REPLICATION_COMPLEX_FORMATIONKEGG_MAPK_SIGNALING_PATHWAYREACTOME_RECRUITMENT_OF_NUMA_TO_MITOTIC_CENTROSOMESREACTOME_PLATELET_ACTIVATION_TRIGGERSBIOCARTA_HIVNEF_PATHWAYBIOCARTA_AKAP95_PATHWAYREACTOME_PHOSPHORYLATION_OF_THE_APCKEGG_B_CELL_RECEPTOR_SIGNALING_PATHWAY
Late proteins	LCC size = 5M = 7<k> = 64.22<kin> = 0.78<kout> = 1.17pin, out = 0.24	TAB1, IL1RN, IL1B, NAMPT, VDAC1, NCF1, CTTN, RPS24, CD74, TANK, BIRC2, TRAF3, IRAK1, TRADD, RANBP9, CRK, CASP7, NCF2	KEGG_LEISHMANIA_INFECTIONKEGG_APOPTOSISBIOCARTA_IL1R_PATHWAYBIOCARTA_NFKB_PATHWAYKEGG_TOLL_LIKE_RECEPTOR_SIGNALING_PATHWAYBIOCARTA_DEATH_PATHWAYBIOCARTA_HIVNEF_PATHWAYKEGG_NOD_LIKE_RECEPTOR_SIGNALING_PATHWAYKEGG_RIG_I_LIKE_RECEPTOR_SIGNALING_PATHWAYBIOCARTA_TNFR2_PATHWAYBIOCARTA_MITOCHONDRIA_PATHWAYBIOCARTA_CASPASE_PATHWAYBIOCARTA_STRESS_PATHWAYREACTOME_APOPTOSISBIOCARTA_TOLL_PATHWAYREACTOME_GENES_INVOLVED_IN_APOPTOTIC_CLEAVAGE_OF_CELLULAR_PROTEINSREACTOME_APOPTOTIC_EXECUTION_PHASEKEGG_MAPK_SIGNALING_PATHWAYKEGG_SMALL_CELL_LUNG_CANCERREACTOME_TOLL_RECEPTOR_CASCADES
