# Supplementary material for: Endophenotype Network Models: Common Core of Complex Diseases
Source: Sci Rep. 2016 Jun 9;6:27414. doi: 10.1038/srep27414 (PMC4899691; doi:10.1038/srep27414)
Supplement: Supplementary Information [file srep27414-s1.pdf]

Ms. # SREP-2015-26291C

Endophenotype Network Models: Common Core of Complex Diseases

Authors: Susan Dina Ghiassian[1, 3], Jörg Menche[1, 3,5], Daniel Chasman[7], Franco Giulianini, Ruisheng Wang[2], Piero Ricchiuto[8], Masanori Aikawa[8,9], Hiroshi Iwata[8], Christian Muüller [10, 11], Tania Zeller[10 ,11], Amitabh Sharma[1, 3,4], Philipp Wild[11, 12, 13], Karl Lackner[11, 14], Sasha Singh[8], Paul M Ridker[7], Stefan Blankenberg[10, 11], Albert-László Barabási[1, 3,4,5,6], and Joseph Loscalzo[2,]*

Supplementary Information:

Human Interactome

We only consider direct physical protein-protein interactions with reported experimental evidence. For this purpose, we consolidated several data sources including:

(i) Regulatory interactions: We used the TRANSFAC database that lists experimentally derived regulatory interactions. The resulting network, in which nodes represent transcription factors and edges (connections) represent physical binding to regulatory elements, consists of 774 transcription factors and genes connected with 1,335 interactions.

(ii) Binary interactions: We combined several yeast-two-hybrid (Y2H) high-throughput datasets with binary interactions from IntAct and MINT databases. Together these data sources yield 28,653 interactions between 8,120 proteins.

(iii) Literature-curated interactions: These interactions, typically obtained by low throughput experiments, are manually curated from the literature. We used IntAct, MINT, BioGRID, and HPRD resulting in 88,349 interactions between 11,798 proteins.

(iv) Metabolic enzyme-coupled interactions: Two enzymes are assumed to be coupled if they

share adjacent reactions in the KEGG and BIGG databases. In total, we used 5,325 such metabolic links between 921 enzymes.

(v) Protein complexes: Protein complexes are single molecular units that integrate multiple gene products. The CORUM database is a collection of mammalian complexes derived from a variety of experimental tools, from co-immunoprecipitation to co-sedimentation and ion exchange chromatography. In total, CORUM yields 2,837 complexes with 2,069 proteins connected by 31,276 links.

(vi) Kinase network (kinase-substrate pairs): Protein kinases are important regulators in different biological processes, such as signal transduction. PhosphositePlus provides a network of 1,843 kinases and substrates connected through 6,066 interactions.

(vii) Signaling interactions: The dataset from provides 32,706 interactions between 6,339 proteins that integrates several sources, derived from both high-throughput screens and literature curation, into a directed network in which cellular signals are transmitted by protein-protein interactions. Note that in our analysis, we do not take into account the direction of these interactions.

(viii) Liver-specific interactions: We also include liver-specific protein-protein interaction data as many of the mediator proteins in inflammation, thrombosis, and fibrosis are synthesized in the liver.

The union of all interactions obtained from (i)-(vii) yields a network of 13,681 proteins that are interconnected by 144,414 physical interactions. The network has a power-law degree distribution with a few hubs and a substantial number of low-degree nodes, and shows other typical characteristics observed previously in biological networks, such as high clustering and short path lengths.

The effect of biased studies of human interactome on topological properties of disease genes

Current maps of the human interactome are prone to investigative biases (*43, 44*). Since disease genes are

typically the particular focus of experimental research, it is often observed that they have more established interaction partners and, therefore, higher degree in the network.

Here, we aim to explore how biased studies of the human interactome may affect observing significant clustering of seed genes in the network. To quantify the extent to which the observed topological properties of disease proteins is due to these biased studies, we repeated our analysis on an unbiased, Y2H high-throughput subset of the human interactome.

The raw observation suggests that proteins show much smaller clustering effect on unbiased protein-protein interaction network. However, note that the interaction among substantial number of seed genes has not yet been examined in current high-throughput maps (Y2H network), and, therefore, the conclusion of such observation requires more attention.

As mentioned above, due to limited search space (number of proteins examined) and an interaction detection sensitivity of ~10%, the unbiased maps are much sparser than current LCI maps. Thus, observing a smaller clustering effect is, indeed, expected and can be explained by the incompleteness of the current maps of unbiased interactome. Moreover, LCI is not limited to protein-protein interactions and includes interactions from several sources such as metabolic, regulatory, etc.

To show that the observed topological properties of disease proteins on unbiased maps is, indeed, due to the incompleteness of the network (and not solely due to the biased nature of studies), we proceed as follow (Fig. S7*a* for the flowchart):

1. For a fair comparison, we first limit the nodes of our HI to those existing in Y2H. Therefore, we characterized the subnetwork of the full network that contains Y2H network nodes. This subnetwork contains a substantially larger number of edges than the Y2H network. The latter can be viewed as an incomplete but unbiased subset of this subnetwork.
2. Next, we check whether the differences are significant or expected by chance. We randomly

remove (“prune”) links from this subnetwork until we reach the same number of links as in the Y2H network. In parallel, we try to keep the degree of the nodes preserved as in the Y2H network.

Our analysis shows that the observed low clustering of seed genes in unbiased maps lies within the expected range drawn by randomly pruned events (Fig, S7*b*). Therefore, low clustering of disease proteins can, to a great extent, be explained by the incompleteness of the network.

DIAMOnD Robustness and topological validation of module size

To check the robustness of the module-finding methodology towards false positives and genes mis-annotations we performed the so-called N-1 analysis where N is the original number of seed genes. In this analysis, we remove one seed each time and expand the neighborhood of N-1 seeds iteratively. At each iteration, we measure the overlap of the detected genes between the original (N seeds) and the trial (N-1 seeds) sets. This procedure allows us to define an upper threshold and limit for the size of the final module above which the outcome of the methodology is sensitive to false positive annotations of seeds.

Figure S2 shows the average overlap of the detected DIAMOnD genes as opposed to the DIAMOnD iteration step. The overlap has been measured between the genes resulting from two different seed sets: the original seeds and N different configurations of trial seeds (each containing N-1 seeds). As shown in the figure the methodology is robust towards small variation of seed genes.

Fully embedded pathways

For pathway analysis, we used version 3.1 of Molecular Signature Database (MSigDB) developed by the Broad Institute, which is an integration of several different databases. Here we use pathways from KEGG, Reactome, and Biocarta. Each pathway is associated with a list of genes for which we calculate their enrichment using Fisher’s exact test. However, there are a few pathways, in which their associated genes

are fully (%100) embedded within our detected modules and are suspected to have a direct role in inflammatory processes (Table S1 and Fig. 2*d*); note that, given the current coverage of the map of human Interactome, proteins belonging to the pathway "Reactome_activation_of_AP1_family_of_transcription_factor" do not directly interact with each other.

Supplementary Figures:

Fig. S1. Genetic association of seed genes as compared to other genes with respect to three cardiovascular biomarkers, CRP, fibrinogen, and sICAM, as well as the specific vascular disease phenotype, VTE, in the inflammatory (pink background, first column), thrombotic (light blue background, second column), and fibrotic (light orange, third column) subnetworks. Seed genes contain more low *p-value* GWAS genes than other genes in the network [red circles, seed genes; green circles, endophenotype module (subnetwork); black circles, rest of network].

Fig. S2. *N*-1 analysis. All modules are robust towards removing one node from the initial set of seed genes. The largest deviation appears as a consequence of removing the gene A2M from the fibrosis seed genes.

Fig. S3. Venn diagram of differentially expressed genes associated with cardiovascular risk factors CRP, fibrinogen, HDL, triglycerides, and APO-A.

Fig. S4. Tree analysis. (**A**) Given a set of nodes, we start by removing them from the HI and recording the number of remaining connected components and their LCC size. Next, we compare this analysis to that of random expectations where same number of nodes is randomly removed from the network. Blue arrows show the observed LCC size and number of connected components of the remaining nodes where (**B**) and (**C**) show a trunk-like and leaf-like behavior of a given set of nodes, respectively.

Fig. S5. Functional similarities of ome-M proteins. The flowchart shows the steps towards calculating functional cohesiveness among ome-M proteins inside and outside detected modules. Detected proteins inside modules are functionally and significantly different from the ome-M proteins.

Fig S6. Confirming the M1-polarization of Thp1 cells using IFNγ**.** IFNγ treatment in THP-1 cells induced the potent pro-inflammatory molecules, including TNFα and IL-1β, the two commonly used markers of M1 macrophages. Each bar represents four independent experiments, where each replicates has three technical repeats. We can see that the expression levels of these molecule typically increase during M1 polarization.

Fig. S7. Studying biased studies of networks in seeds clustering effects. (**A**) The flowchart of fairly comparing seeds clustering effects within curated networks and unbiased networks. (**B**) The observed clustering of seeds within unbiased maps lies within the expected range drawn by randomization.

Supplementary Tables:

Table S1. Fully embedded pathways in different regions of endo-phenotype modules and their associated genes

**Fully embedded pathways**	**Network neighborhood**	**Genes**
BIOCARTA_IL6_PATHWAY	Inflammasome-Thrombosome crosstalk	CSNK2A1, JUN, SRF, HRAS, IL6R, STAT3, PTPN11, IL6ST, RAF1, SHC1, ELK1, MAP2K1, TYK2, JAK1, JAK3, JAK2, CEBPB, GRB2, MAPK3, FOS, IL6, SOS1
BIOCARTA_EXTRINSIC_PATHWAY	Thrombosome specific	F10, TFPI, F2, F2R, FGG, PROS1, PROC, FGA, SERPINC1, FGB, F3, F5, F7
REACTOME_PECAM1_INTERACTIONS	Modules crosstalk	ITGAV, SRC, PTPN11, PLCG1, PECAM1, YES1, FYN, ITGB3, PTPN6, LYN, LCK, INPP5D
REACTOME_ACTIVATION_OF_THE_AP1_FAMILY_OF_TRANSCRIPTION_FACTOR	Modules crosstalk	MAPK9, JUN, MAPK10, MAPK11, MAPK8, MAPK14, ATF2, MAPK3, FOS, MAPK1
BIOCARTA_IGF1_PATHWAY	Inflammasome specific	CSNK2A1, JUN, SRF, HRAS, MAPK8, PTPN11, RAF1, SHC1, ELK1, MAP2K1,I GF1, RASA1, PIK3CA, IGF1R, GRB2, IRS1, MAPK3, FOS, PIK3CG, PIK3R1, SOS1

Table S2. Differentially expressed genes associated with cardiovascular risks and their overlap with endo-phenotype modules

Molecule	#dE genes	Overlap with inflammation module (seeds)	Overlap with thrombosis module (seeds)	Overlap with fibrosis module (seeds)
CRP	479	55 (28)	52 (11)	35 (6)
Fibrinogen	255	20 (8)	19 (3)	15 (2)
APO-A	136	19 (11)	17 (3)	15 (2)
APO-B	9	1 (0)	0 (0)	1 (0)
HDL	216	34 (17)	30 (7)	24 (4)
LDL	3	1 (1)	1 (0)	0 (0)
Triglyceride	57	8 (4)	8 (2)	4 (1)

Table S3. Number of diseases significantly enriched with endo-phenotype modules.

	**Seeds**	**Module**
Inflammation	95	117
Thrombosis	83	116
Fibrosis	77	99

Table S4. Significantly enriched diseases in module-specific regions

**Cross-talk**		**Inflammasome-specific**	**Thrombosome-specific**
arthritis<br>autoimmune-diseases<br>autoimmune-diseases-of-the-nervous-system<br>bile-duct-diseases<br>bone-diseases<br>bone-marrow-diseases<br>cardiovascular-abnormalities<br>cardiovascular-diseases<br>cerebrovascular-disorders<br>colitis<br>colonic-diseases<br>colorectal-neoplasms<br>congenital-abnormalities<br>connective-tissue-diseases<br>crohn-disease<br>demyelinating-	intestinal-neoplasms<br>joint-diseases<br>kidney-diseases<br>kidney-neoplasms<br>leukemia<br>liver-diseases<br>lung-diseases<br>lymphatic-diseases<br>lymphoproliferative-disorders<br>male-urogenital-diseases<br>metabolic-diseases<br>multiple-sclerosis<br>musculoskeletal-abnormalities<br>musculoskeletal-diseases<br>myeloproliferative-disorders<br>neoplasms<br>neoplasms-by-	arteriosclerosis<br>central-nervous-system-diseases<br>death-sudden<br>death-sudden-cardiac<br>heart-arrest<br>heart-defects-congenital<br>muscular-disorders-atrophic<br>neuroectodermal-tumors<br>neuroendocrine-tumors<br>skin-diseases-genetic<br>stomatognathic-diseases<br><br>**Fibrosome-specific**<br>adenocarcinoma<br>arrhythmias-cardiac<br>genital-diseases-male	abnormalities,-multiple<br>anemia,-aplastic<br>bone-diseases,-developmental<br>breast-neoplasms<br>carcinoma,-renal-cell<br>charcot-marie-tooth-disease<br>colitis,-ulcerative<br>collagen-diseases<br>congenital,-hereditary,-and-neonatal-diseases-and-abnormalities<br>death,-sudden<br>demyelinating-autoimmune-diseases,-cns<br>diabetes-mellitus,-type-1

diseases<br>diabetes-mellitus<br>digestive-system-diseases<br>digestive-system-neoplasms<br>endocrine-system-diseases<br>female-urogenital-diseases<br>female-urogenital-diseases-and-pregnancy-complications<br>gastroenteritis<br>gastrointestinal-diseases<br>glucose-metabolism-disorders<br>gonadal-disorders<br>heart-diseases<br>hematologic-diseases<br>hemic-and-lymphatic-diseases<br>hemorrhagic-disorders	histologic-type<br>neoplasms-by-site<br>nephritis<br>nervous-system-diseases<br>nervous-system-malformations<br>neurodegenerative-diseases<br>nutritional-and-metabolic-diseases<br>peripheral-nervous-system-diseases<br>pigmentation-disorders<br>psoriasis<br>respiratory-tract-diseases<br>rheumatic-diseases<br>skin-and-connective-tissue-diseases<br>skin-diseases<br>urogenital-neoplasms<br>urologic-diseases<br>vascular-diseases	genital-neoplasms-male<br>glioma<br>lymphoma-non-hodgkin<br>macular-degeneration<br>otorhinolaryngologic-diseases<br>prostatic-diseases<br>prostatic-neoplasms	genetic-diseases,-inborn<br>genetic-diseases,-x-linked<br>genital-diseases,-female<br>heart-defects,-congenital<br>infant,-newborn,-diseases<br>leukemia,-lymphoid<br>leukemia,-myeloid<br>lung-diseases,-obstructive<br>lupus-erythematosus,-systemic<br>lymphoma,-non-hodgkin<br>neoplasms,-glandular-and-epithelial<br>neoplastic-syndromes,-hereditary<br>skin-diseases,-papulosquamous<br>urologic-neoplasms

immune-system-diseases immunoproliferative-disorders inflammatory-bowel-diseases intestinal-diseases			

Table S5. Topological network properties of endo-phenotype modules

**Network Region**	**Betweenness centrality**	**Network degree**
Inflammation seeds	0.00059	36.92
Inflammation DIAMOnD nodes	0.0015	87.16
Inflammation module	0.0011	62.26
Thrombosis seeds	0.000037	28.28
Thrombosis DIAMOnD nodes	0.0013	80.11
Thrombosis module	0.0012	70.69
Fibrosis seeds	0.00059	38.34
Fibrosis DIAMOnD nodes	0.0015	85.40
Fibrosis module	0.0014	78.62
Network	0.00019	21.11

Table S6. Topological and biological properties of early and late proteins characterized by confidence level criterion (b): *p*-value<0.05

**p<0.05**	**Statistics**	**Proteins**	**Top 20 enriched pathways**
Early proteins	#proteins = 47<br>M = 36<br>LCC size = 23<br><k> = 64.57<br><kin> = 1.53<br><kout> = 2.76<br>*P*-value = 0.04	STMN1, VAV3, ITGA3, CARD9, GNA12, IFNGR1, VCL, RASA1, PARP1, CD36, SCARB1, CSNK2A1, PRKDC, CD9, LRPPRC, HSPB1, PTPN2, TOP2A, CLTC, CABIN1, CD58, MTHFD1, PIK3CG, CBS, PTGS1, CD22, TNPO1, CAD, DHFR, PEBP1, GPX1, AKT2, PON2, ROCK2, CD2AP, CCNB1,	REACTOME_HEMOSTASIS<br>REACTOME_FORMATION_OF_PLATELET_PLUG<br>KEGG_REGULATION_OF_ACTIN_CYTOSKELETON<br>REACTOME_PLATELET_ACTIVATION<br>KEGG_FOCAL_ADHESION<br>REACTOME_GAP_JUNCTION_DEGRADATION<br>BIOCARTA_PTC1_PATHWAY<br>BIOCARTA_SRCRPTP_PATHWAY<br>KEGG_PROGESTERONE_MEDIATED_OOCYTE_MATURATION<br>REACTOME_CYCLIN_A1_ASSOCIATED_EVENTS_DURING_G2_M_TRANSITION<br>KEGG_CHEMOKINE_SIGNALING_PATHWAY<br>REACTOME_PLATELET_ACTIVATION_TRIGGERS<br>BIOCARTA_HIVNEF_PATHWAY<br>BIOCARTA_IGF1_PATHWAY<br>REACTOME_GAP_JUNCTION_TRAFFICKING<br>REACTOME_COLLAGEN_MEDIATED_ACTIVATION_CASCADE

		DAB2, CALM1, BRAF, MYO6, CASP8, IQGAP1, CDK1, CDK9, CDK7, CSK, RPS27A	BIOCARTA_INSULIN_PATHWAY<br>KEGG_GLIOMA<br>KEGG_NEUROTROPHIN_SIGNALING_PATHWAY<br>BIOCARTA_CELLCYCLE_PATHWAY
Late proteins	#proteins = 55<br>M = 159<br>LCC size = 47<br><k> = 107.6<br><kin> = 5.78<br><kout> = 2.36<br>*P*-value = $1.7e^{-4}$	TAB1, IL1RN, IL1B, NAMPT, AHCY, HMGB1, VDAC1, VDAC2, NCF1, LIMA1, CTTN, RPS24, KRAS, TRAF3, IRAK1, TRADD, CSNK2B, PAK2, CASP4, VLDLR, VIM, HSPA9, HSPA8, TLR2, ABI1, HSP90AA1, HSPD1, PPM1B, ACTN4, RDX,	REACTOME_HEMOSTASIS<br>REACTOME_FORMATION_OF_PLATELET_PLUG<br>KEGG_REGULATION_OF_ACTIN_CYTOSKELETON<br>REACTOME_PLATELET_ACTIVATION<br>KEGG_FOCAL_ADHESION<br>REACTOME_GAP_JUNCTION_DEGRADATION<br>BIOCARTA_PTC1_PATHWAY<br>BIOCARTA_SRCRPTP_PATHWAY<br>KEGG_PROGESTERONE_MEDIATED_OOCYTE_MATURATION<br>REACTOME_CYCLIN_A1_ASSOCIATED_EVENTS_DURING_G2_M_TRANSITION<br>KEGG_CHEMOKINE_SIGNALING_PATHWAY<br>REACTOME_PLATELET_ACTIVATION_TRIGGERS<br>BIOCARTA_HIVNEF_PATHWAY<br>BIOCARTA_IGF1_PATHWAY

		REL, ALOX5, EDF1, CD74, GRB2, NFKB2, GNB2L1, TANK, ENO1, BIRC2, HCLS1, RAN, EIF3E, RANBP9, MAPK13, CRK, ASAP1, LGALS3, CASP7, NCF2, PTPN12, HNRNPA1, RPL22, HSPA5, PAFAH1B1	REACTOME_GAP_JUNCTION_TRAFFICKING REACTOME_COLLAGEN_MEDIATED_ACTIVATION_CASCADE BIOCARTA_INSULIN_PATHWAY KEGG_GLIOMA KEGG_NEUROTROPHIN_SIGNALING_PATHWAY BIOCARTA_CELLCYCLE_PATHWAY

Table S7. Topological and biological properties of early and late proteins characterized by confidence level criterion (c): FC>1.5

FC < 1.5	Statistics	Proteins	Top 20 enriched pathways
Early proteins	#proteins = 67<br>M = 85<br>LCC size = 36<br><k> = 72.81<br><kin> = 2.54<br><kout> = 1.37<br>*P*-value = $4.47e^{-3}$	STMN1, VAV3, CALU, DNAJB1, ITGAL, ITGA4, ITGA3, RPS6KA3, CARD9, GNA12, CAV1, IFNGR1, GSTM1, PTGES3, RASA1, PARP1, CD36, SCARB1, CSNK2A1, PTGS1, CD22, EIF3M, TNPO1, TFRC, DHFR, PPP1CA, GAPDH, PEBP1, EPHX1, AGT, ETS1, AHSG, GPX1, AKT2, RPS27A, CD63, CSNK2A2, GC, PIK3CB, CCNB1, EIF4A2, CALM1, ALB, GNAI2, PGRMC2, GGT1, CDK1, CDK9, CDK7, HLA-B, CSK, HCK, TBXAS1, PRKDC, CD9, LRPPRC, IFI30, CASP3, HSPB1, HSPA14, PLA2G4A, MYL6, TOP2A, CLTC, CABIN1, TXN, CD58	REACTOME_HEMOSTASIS<br>REACTOME_FORMATION_OF_PLATELET_PLUG<br>REACTOME_PLATELET_ACTIVATION<br>REACTOME_PLATELET_ACTIVATION_TRIGGERS<br>KEGG_ARACHIDONIC_ACID_METABOLISM<br>KEGG_PROGESTERONE_MEDIATED_OOCYTE_MATURATION<br>REACTOME_PLATELET_DEGRANULATION<br>KEGG_HEMATOPOIETIC_CELL_LINEAGE<br>REACTOME_SIGNALING_IN_IMMUNE_SYSTEM<br>REACTOME_CELL_SURFACE_INTERACTIONS_AT_THE_VASCULAR_WALL<br>KEGG_REGULATION_OF_ACTIN_CYTOSKELETON<br>REACTOME_PROSTANOID_HORMONES<br>BIOCARTA_PTC1_PATHWAY<br>BIOCARTA_SRCRPTP_PATHWAY<br>BIOCARTA_AKAP95_PATHWAY<br>KEGG_MAPK_SIGNALING_PATHWAY<br>KEGG_NATURAL_KILLER_CELL_MEDIATED_CYTOTOXICITY<br>KEGG_FOCAL_ADHESION<br>REACTOME_CYCLIN_A1_ASSOCIATED_EVENTS_DURING_G2_M_TRANSITION<br>REACTOME_HORMONE_BIOSYNTHESIS

Late proteins	#proteins = 42<br>LCC size = 8<br>M = 21<br><k> = 63.88<br><kin> = 1<br><kout> = 2.19<br>*P*-value = $6.14e^{-3}$	MYL12A, TANK, FSCN1, BIRC2, TRAF3, IRAK1, TRADD, RHOC, RELB, JUN, MX1, TGM2, RANBP9, MAPK9, CRK, HRAS, CASP10, CASP7, NCF2, IFIH1, SPP1, LPL, TAB1, IL1RN, IL1B, DOK1, NAMPT, RPS6KA5, GAB2, MARCKS, CAMK2G, RPS13, VDAC1, NCF1, TIMP3, FYB, ARHGAP17, CTTN, RPS24, IRF5, CD74, ITGB3	REACTOME_HEMOSTASIS<br>REACTOME_FORMATION_OF_PLATELET_PLUG<br>REACTOME_PLATELET_ACTIVATION<br>REACTOME_PLATELET_ACTIVATION_TRIGGERS<br>KEGG_ARACHIDONIC_ACID_METABOLISM<br>KEGG_PROGESTERONE_MEDIATED_OOCYTE_MATURATION<br>REACTOME_PLATELET_DEGRANULATION<br>KEGG_HEMATOPOIETIC_CELL_LINEAGE<br>REACTOME_SIGNALING_IN_IMMUNE_SYSTEM<br>REACTOME_CELL_SURFACE_INTERACTIONS_AT_THE_VASCULAR_WALL<br>KEGG_REGULATION_OF_ACTIN_CYTOSKELETON<br>REACTOME_PROSTANOID_HORMONES<br>BIOCARTA_PTC1_PATHWAY<br>BIOCARTA_SRCRPTP_PATHWAY<br>BIOCARTA_AKAP95_PATHWAY<br>KEGG_MAPK_SIGNALING_PATHWAY<br>KEGG_NATURAL_KILLER_CELL_MEDIATED_CYTOTOXICITY<br>KEGG_FOCAL_ADHESION<br>REACTOME_CYCLIN_A1_ASSOCIATED_EVENTS_DURING_G2_M_TRANSITION<br>REACTOME_HORMONE_BIOSYNTHESIS

Table S8. Case and control Sample sizes and biomarker level ranges

Molecule	Case sample size High risk range	Control sample size Low risk range
CRP	375 (>3 mg/l)	372 (<1 mg/l)
Fibrinogen	324 (>402 mg/dl)	325 (<302 mg/dl)
APO-A	329 (<1.46 g/l)	325 (>1.86 g/l)
APO-B	328 (>1.20 g/l)	330 (<0.85 g/l)
HDL	328 (<45 mg/dl)	346 (>66 mg/dl)
LDL	316 (>164 mg/dl)	318 (<115 mg/dl)
Triglyceride	325 (>153 mg/dl)	327 (<80 mg/dl)

Additional files not embedded into this document:

Data files S1 and S2

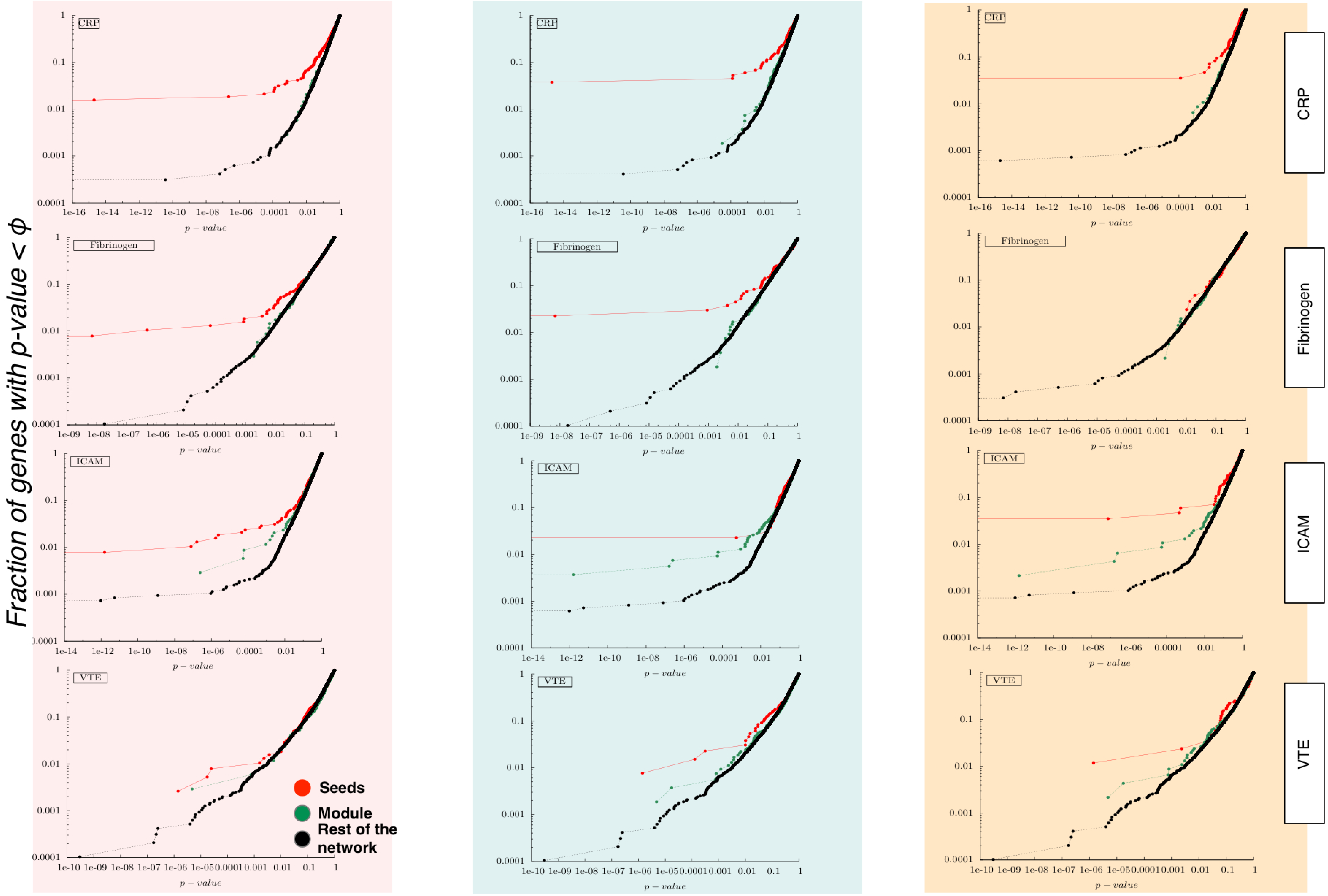
Fraction of genes with p-value < Φ
CRP
Fibrinogen
ICAM
VTE
p − value
Seeds
Module
Rest of the network

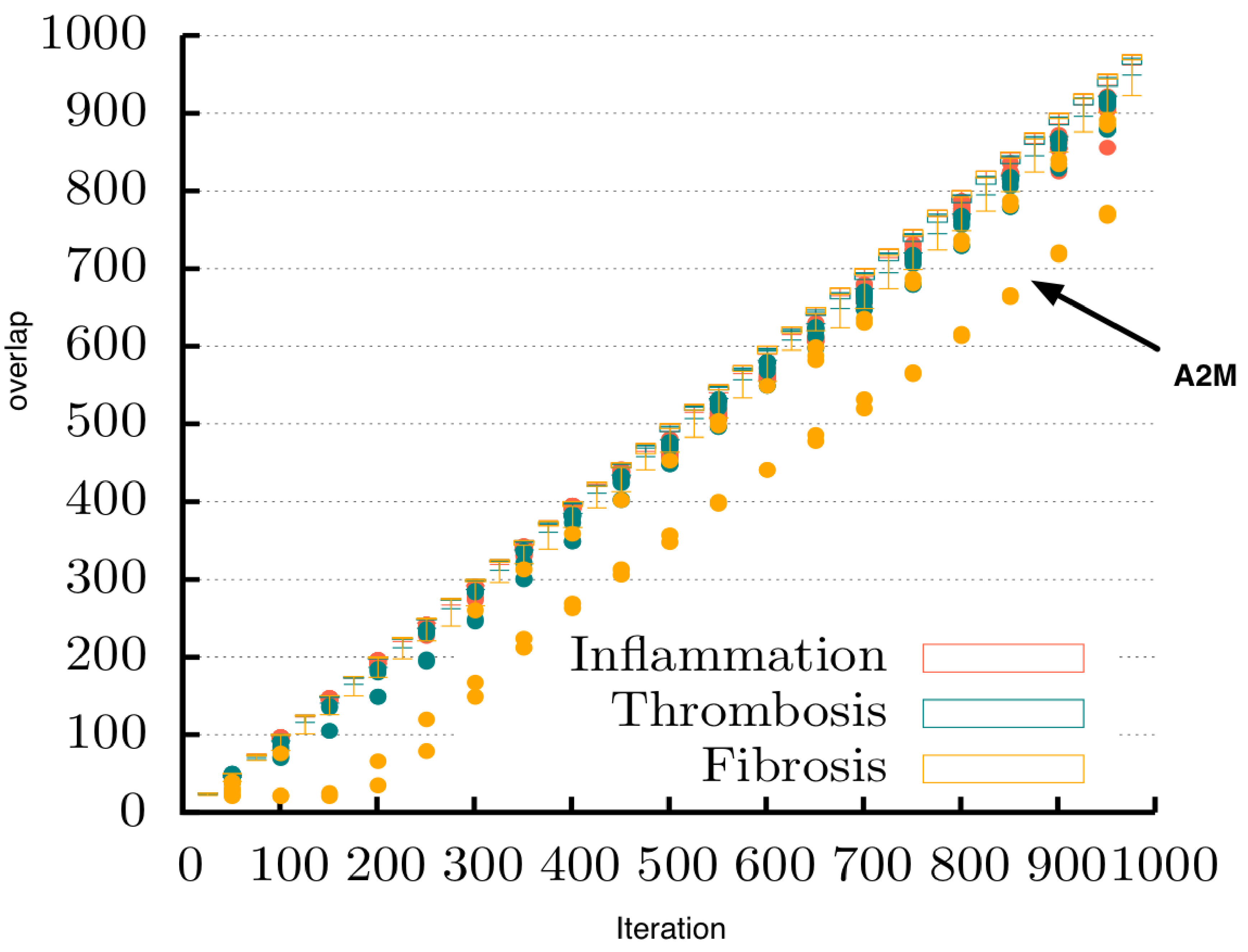
1000
900
800
700
600
500
400
300
200
100
0
overlap
0
100
200
300
400
500
600
700
800
900
1000
Iteration
A2M
Inflammation
Thrombosis
Fibrosis

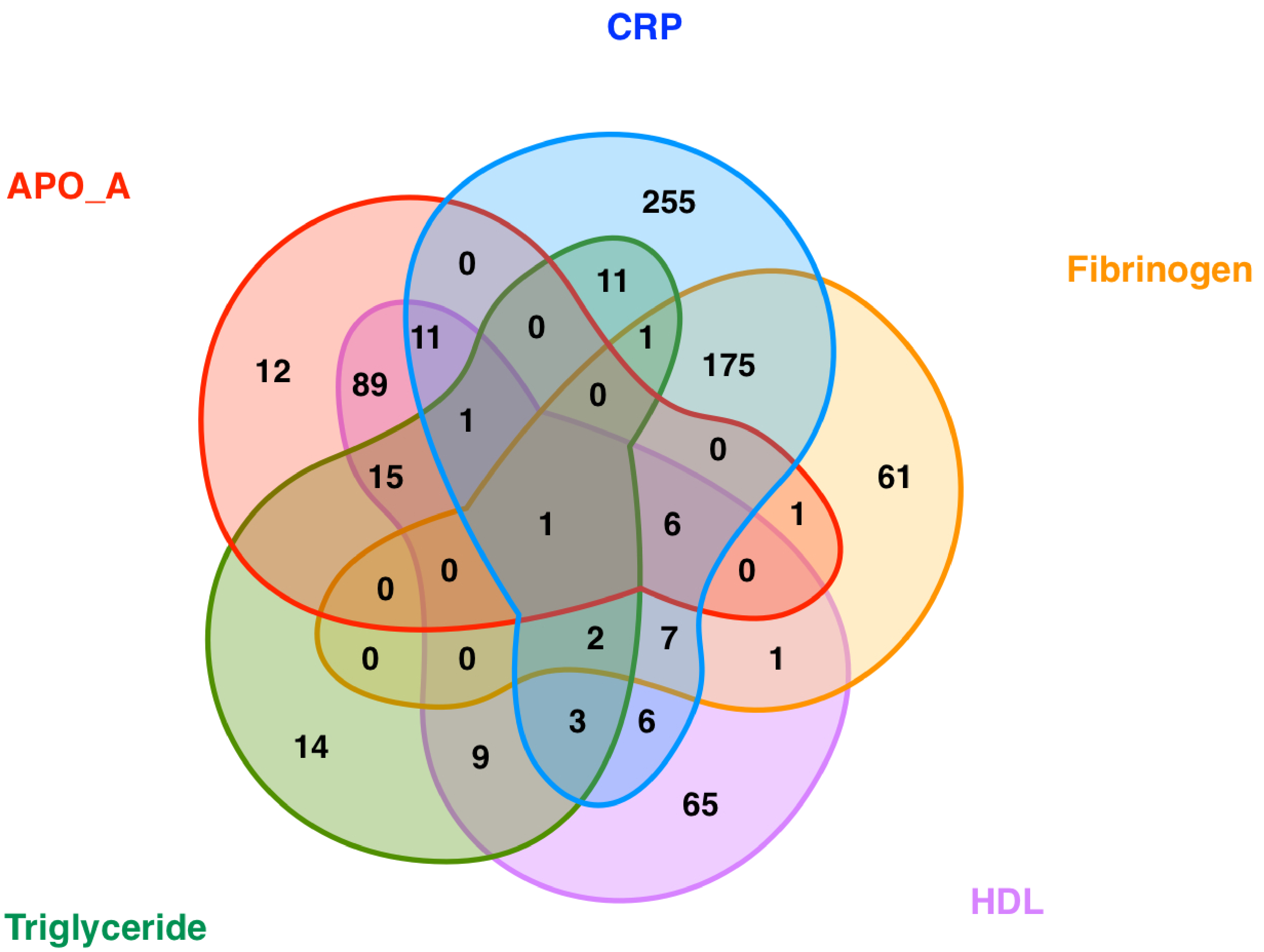

CRP
APO_A
Fibrinogen
Triglyceride
HDL
255
0
11
11
0
1
12
89
1
0
175
1
0
15
1
6
61
1
0
0
0
2
7
1
0
0
3
6
14
9
65

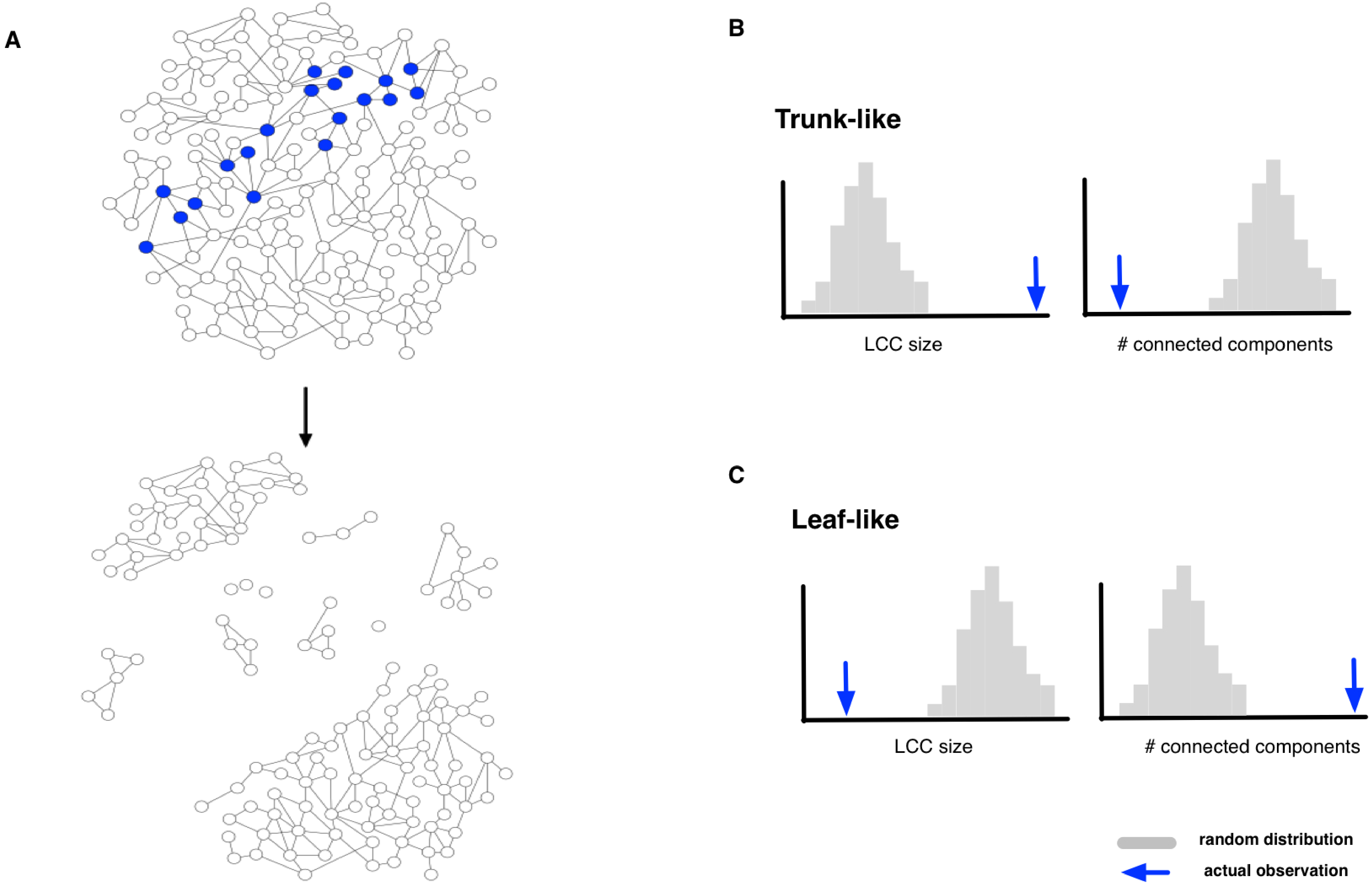
A
B
Trunk-like
LCC size
connected components
C
Leaf-like
LCC size
connected components
random distribution
actual observation

Protein set	Enriched pathways
m-protein inside modules #431	P1,P2,..,Pn
m-protein subset 1	P1,P2,..,Pn1
m-protein subset 2	P1,P2,..,Pn2
m-protein subset 3	P1,P2,..,Pn3
m-protein subset 100	P1,P2,..,Pn100

Calculate functional similarity w all sets below

Calculate functional similarity btw any two combinations

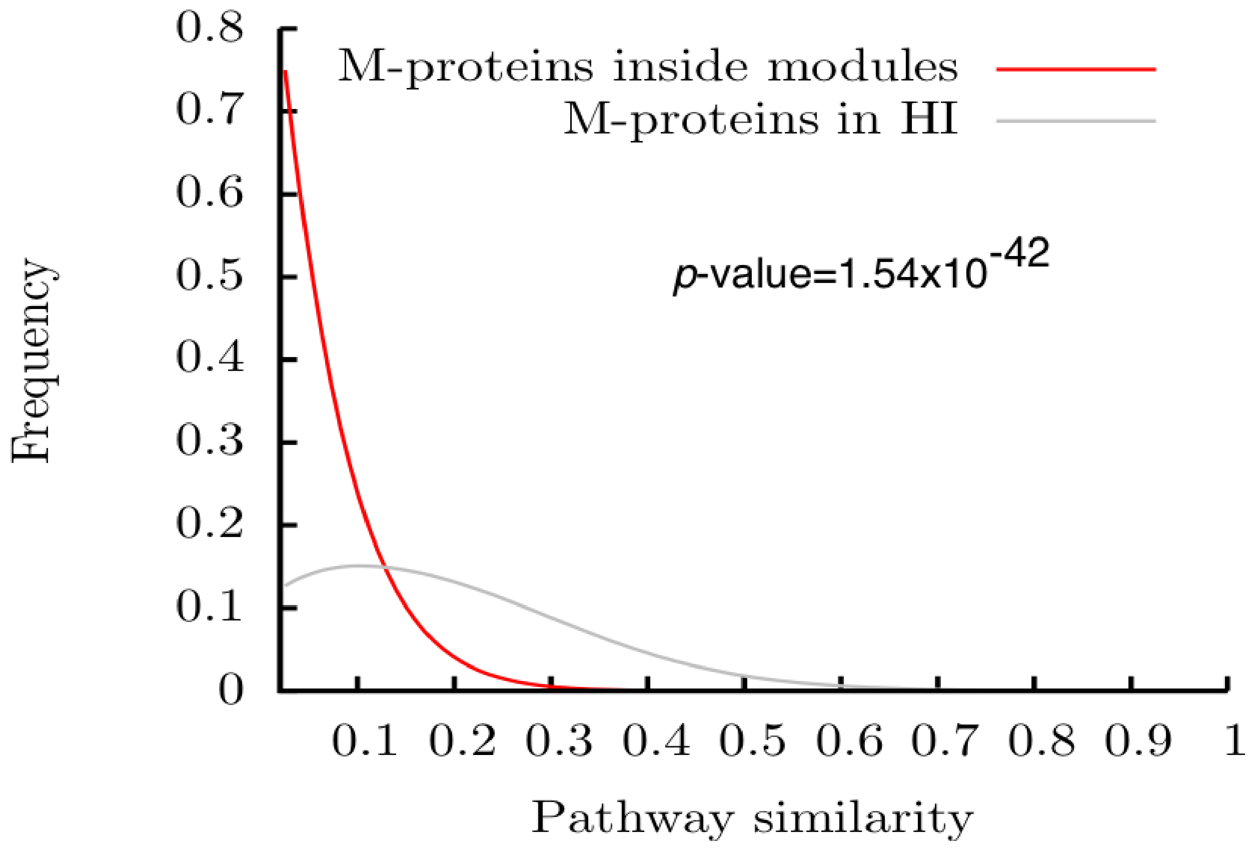

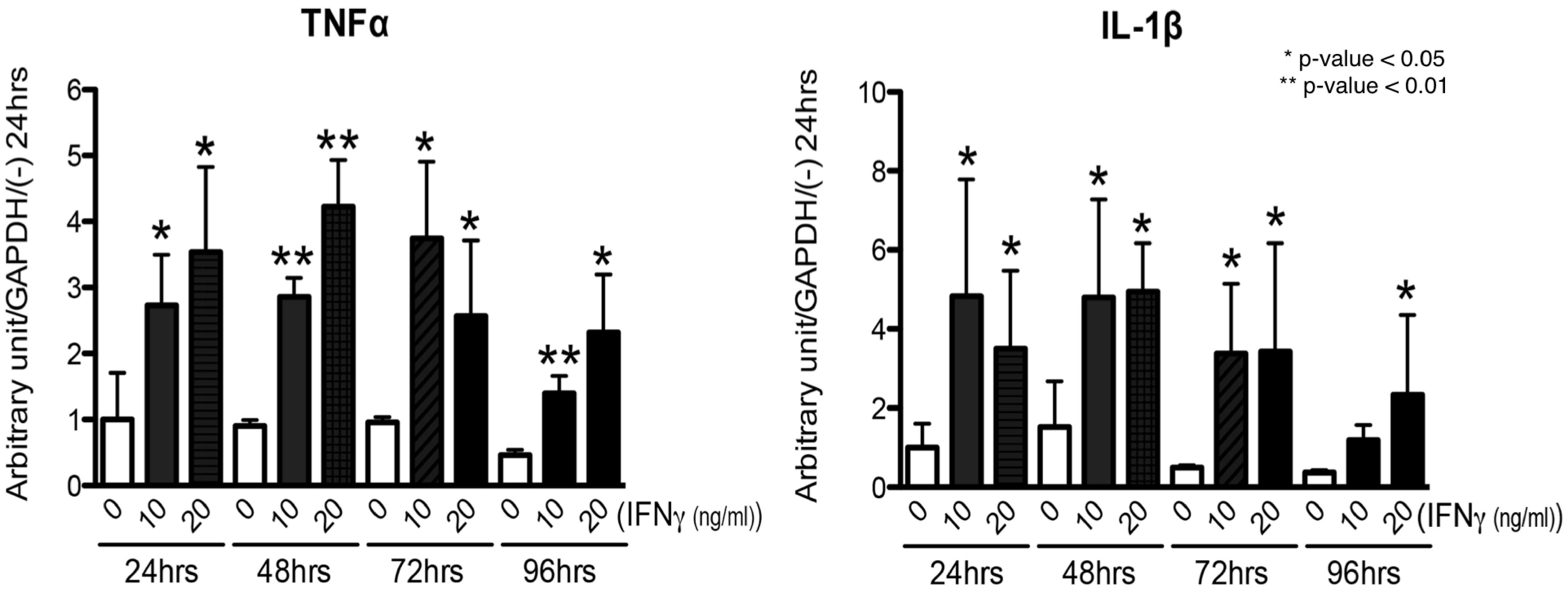
TNFα
Arbitrary unit/GAPDH/(-) 24hrs
0
1
2
3
4
5
6
*
*
**
**
*
*
**
*
0 10 20 0 10 20 0 10 20 0 10 20 (IFNγ (ng/ml))
24hrs
48hrs
72hrs
96hrs
IL-1β
* p-value < 0.05
** p-value < 0.01
Arbitrary unit/GAPDH/(-) 24hrs
0
2
4
6
8
10
*
*
*
*
*
*
*
0 10 20 0 10 20 0 10 20 0 10 20 (IFNγ (ng/ml))
24hrs
48hrs
72hrs
96hrs

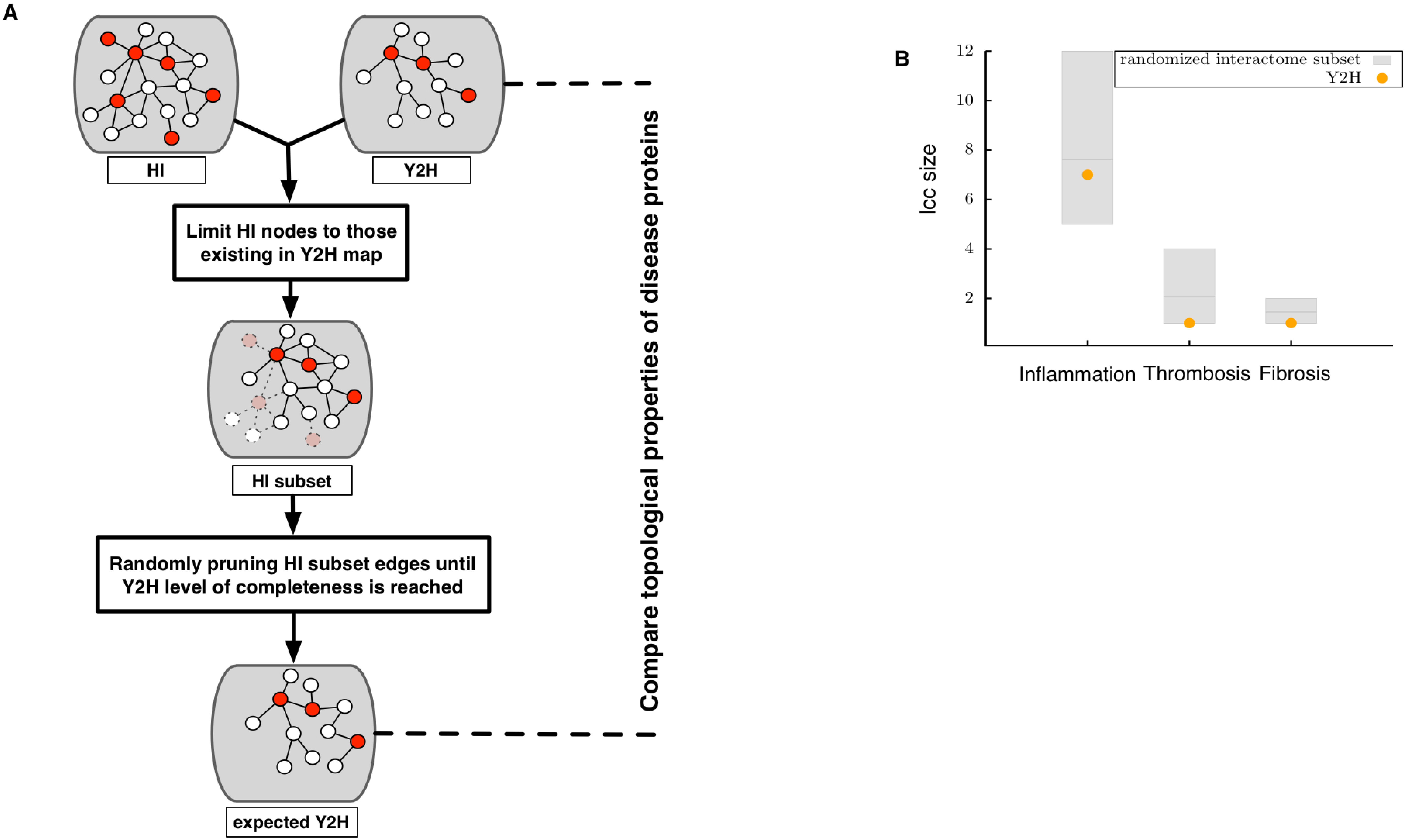
A
HI
Y2H
Limit HI nodes to those existing in Y2H map
HI subset
Randomly pruning HI subset edges until Y2H level of completeness is reached
expected Y2H
Compare topological properties of disease proteins
B
randomized interactome subset
Y2H
lcc size
12
10
8
6
4
2
Inflammation
Thrombosis
Fibrosis
